# The impact of nursing risk management on quality of life, complications, nursing care satisfaction in patients undergoing percutaneous coronary intervention

**DOI:** 10.12669/pjms.42.8.16632

**Published:** 2026-08

**Authors:** Ying Zhang, Zhi Chen, Mengying Gao

**Affiliations:** 1 Ying Zhang, Department of Cardiology, The Second Hospital of Hebei Medical University, Shijiazhuang, Hebei Province 050000, P.R. China; 2 Zhi Chen Medical Equipment Emergency Management Center, Hebei Provincial People’s Hospital, Shijiazhuang, Hebei Province 050000, P.R. China; 3 Mengying Gao, Department of Cardiology, The Second Hospital of Hebei Medical University, Shijiazhuang, Hebei Province 050000, P.R. China

**Keywords:** Coronary heart disease, Nursing risk management, Nursing care satisfaction, Percutaneous coronary intervention

## Abstract

**Objective:**

To explore the impact of nursing risk management on quality of life (QoL), complications and nursing care satisfaction in patients undergoing percutaneous coronary intervention (PCI).

**Methodology:**

A retrospective analysis was conducted of 156 patients with coronary heart disease (CHD) who underwent PCI at the Second Hospital of Hebei Medical University from September 2024 to October 2025. Among them, 78 patients receiving nursing risk management were matched 1:1 with the cohort receiving routine nursing care. QoL, length of hospital stay (LOS), incidence of complications, and nursing care satisfaction were compared between the two groups.

**Results:**

The QoL score of the observation group was significantly higher than that of the control group (P<0.05). The LOS in the observation group was shorter, and the incidence rates of MACE, abdominal distension, irritability, and insomnia were significantly lower in the observation group than in the control group (P<0.05). The nursing care satisfaction rate of the observation group (91.0%) was significantly higher than that of the control group (78.2%) (P<0.05).

**Conclusion:**

Implementing nursing risk management in CHD patients undergoing PCI is associated with improved postoperative QoL, shortened LOS, reduced incidence of postoperative complications, and enhanced nursing care satisfaction.

## INTRODUCTION

Coronary heart disease (CHD) is the leading single cause of mortality and disability-adjusted life years (DALYs) lost worldwide, with an estimated 8.99 million total deaths globally in 2021.^1^,^2^ Percutaneous coronary intervention (PCI) is currently a primary clinical treatment for CHD due to its minimal invasiveness, remarkable efficacy, and rapid recovery.^3^ However, as an invasive procedure, PCI carries potential risks including vascular injury, infection, contrast agent allergy, abdominal distension, irritability, insomnia, and major adverse cardiovascular events (MACE).^4^ Studies have shown that postoperative management of patients undergoing PCI plays a crucial role in ensuring long-term rehabilitation and preventing recurrent MACE.^3^,^4^

The traditional nursing models are primarily disease-centered, emphasizing routine nursing procedures and condition monitoring; they lack systematic assessment, prediction, and targeted intervention of nursing risks, which limits their effectiveness in preventing potential hazards in clinical practice.^5^,^6^ This leads to a persistently high incidence of postoperative complications and limited improvement in nursing care satisfaction.^7^ In recent years, nursing risk management has gradually become a research focus in clinical nursing.^8^ By identifying, evaluating, and analyzing potential risk factors in nursing work, formulating targeted prevention and control measures, and optimizing nursing processes, nursing risk management enables proactive intervention to reduce adverse events and improve nursing quality.^9^

Currently, reports on nursing risk management in PCI patients are limited; there are differences in intervention protocols and outcomes across studies, and the effects of improving postoperative quality of life and preventing and controlling complications remain underexplored.^8^,^9^ This study aimed to compare the impact of nursing risk management and traditional nursing in reducing surgical complications and improving nursing care satisfaction.

## METHODOLOGY

This retrospective analysis included medical records of patients with coronary heart disease who underwent PCI in the Second Hospital of Hebei Medical University from September 2024 to October 2025. Based on differences in nursing regimens, patients who received nursing risk management (Observation group) were matched 1:1 using nearest-neighbor matching with those who received routine nursing care (Control group) on gender, age, disease type, hypertension, diabetes mellitus, and the number of diseased coronary vessels.

### Ethical approval:

The study was approved by the Research Ethics Committee of the Second Hospital of Hebei Medical University (Approval letter No. 2026-R132; dated January 23, 2026). Informed consent was waived due to the retrospective study design.

### Inclusion Criteria:

- Age ≥ 18 years old.
- Diagnosed with coronary heart disease by coronary angiography and scheduled for PCI.
- Normal coagulation function.
- First-time PCI and no history of cardiovascular surgery.
- Complete clinical data.

### Exclusion Criteria:

- Patients with severe physical or mental diseases.
- Patients with concomitant severe hepatic and renal insufficiency.
- Patients with malignant tumors.
- Pregnant or lactating women.

### Routine nursing care:

Before surgery, patients were assisted in completing admission assessments and various preoperative examinations. They received education regarding the surgical procedure, preoperative fasting, and fluid restriction. Preparations for surgical supplies were made, and venous access was established.

During surgery, nurses assisted patients in maintaining a comfortable position, performed continuous electrocardiographic monitoring, closely observed vital signs and intraoperative responses, and cooperated with physicians to ensure the smooth completion of the procedure.

After surgery, continuous electrocardiographic monitoring was conducted for 24–48 hours. The puncture site was observed for bleeding or swelling, and the condition of the affected limb was monitored. Vital signs were regularly assessed, medications were administered as prescribed, and patients received guidance on diet, activity, and a six month post-discharge follow-up. Routine nursing care was provided and all nursing activities were documented. No additional nursing risk management–related interventions were implemented.

### Nursing risk management model:

- A nursing risk management team was established, with the head nurse as the leader and attending physicians, primary nurses, and other staff as members. The team organized special discussions on potential risks during coronary heart disease interventional surgery and formulated preventive measures for nursing-related risks.
- Targeted perioperative nursing was implemented. 1) Before surgery, nurses strengthened communication and conducted face-to-face talks with patients to explain the importance and process of interventional surgery, thereby enhancing patients’ confidence in the surgery and encouraging their active cooperation in treatment. 2) During surgery, nurses intensified monitoring of blood pressure and heart rate, informed patients that they might feel pain when anesthetic drugs were injected and distending pain when the sheath was inserted, and guided patients to practice deep breathing to relieve tension and anxiety. 3) After surgery, nurses promptly observed changes in patients’ complexion and consciousness, maintained close communication, and performed local massage if needed. Targeted postoperative nursing risk-prevention measures were implemented. For postoperative low back pain, a soft pad was placed under the patient’s waist, and the massage was performed. Patients were guided to alternate between supine and lateral positions and to use relaxation methods, such as listening to music, to divert attention. Sedative drugs were administered when necessary. For insomnia, nurses strengthened psychological counseling for patients, guided their family members to massage their shoulders, waist, and back to relax muscles, and ensured that patients adopted a comfortable sleeping posture. For urinary retention, nurses advised patients to drink more water and avoid soybean milk and milk. Nurses applied hot compresses to promote urination and performed catheterization for patients who still could not urinate spontaneously after taking necessary measures.
- Continuous quality improvement of the nursing risk management model was strengthened. The patients were followed for six months and the nursing team regularly summarized problems that arose during the nursing process, discussed them, formulated preventive measures, and integrated new nursing methods into the nursing plan for the next stage.

### Observational indices:

The following indicators were collected from all patients. The primary outcomes are QoL, complications, and nursing care satisfaction. The secondary outcomes are length of hospital stay (LOS), major adverse cardiac events (MACE).

### Quality of life:

The Seattle Angina Questionnaire (SAQ) was used to evaluate patients’ QoL before and six months after intervention.^10^ SAQ includes five dimensions (angina stability, angina frequency, physical limitation, disease perception (quality of life), and treatment satisfaction) and 19 items. A lower score indicates a higher degree of angina pectoris.

### LOS, MACE, and complications:

MACE including cardiac death, MI, and target-vessel revascularization. Complications including abdominal distension, irritability, low back pain, insomnia, and urinary retention.

### Nursing Care Satisfaction:

Nursing care satisfaction was evaluated using a self-developed, anonymous questionnaire. Nursing care satisfaction questionnaires were distributed and collected by nurses independent of the nursing team before patients were discharged, in order to comprehensively evaluate the medical staff’s nursing skills, adverse reaction rate control, nurse-patient relationship, and service quality. The questionnaire used a 100-point scoring system: 80–100 points indicated very satisfied, 60–79 points generally satisfied, and < 60 points dissatisfied. Nursing satisfaction rate = (number of very satisfied cases + number of generally satisfied cases) / total number of cases × 100%. A higher score indicates greater satisfaction.

### Statistical analysis:

All data analysis was conducted using SPSS 25.0 software (IBM Corp, Armonk, NY, USA). The Shapiro-Wilk test was used to evaluate the normality of the data distribution for continuous variables. Normal distribution data were represented by mean ± standard deviation, an independent sample t-test was used for intergroup comparison, and a paired t-test was used for intragroup comparison before and after intervention. Non-normally distributed data were reported as median and interquartile range. The Mann-Whitney U test was used for intergroup comparisons, and the Wilcoxon signed-rank test for intragroup comparisons. Categorical variables were presented as counts of cases, and the Chi-square test was used for comparison between groups. P<0.05 was considered statistically significant.

## RESULTS

A total of 156 patients were included in this study, 98 males and 58 females. Ages ranged from 35 to 72 years, with a mean of 52.8±8.1 years. The observation and control groups were matched 1:1, with 78 cases in each group. There was no statistically significant difference in baseline characteristics between the two groups (P>0.05; Table-I).

**Table-I:** Comparison of baseline characteristics between two groups.

Baseline characteristics	Observation group (n=78)	Control group (n=78)	Z/χ^2^	P
***Sex, n(%)***			0.988	0.32
Male	46 (59.0)	52 (66.7)		
Female	32 (41.0)	26 (33.3)		
Age (years), mean±SD	52.3±7.9	53.2±8.4	-0.63	0.53
BMI (kg/m²), mean±SD	23.9±2.4	23.3±3.0	1.587	0.115
***Educational attainment, n(%)***			1.292	0.524
Junior high school and below	38 (48.7)	36 (46.2)		
High school	24 (30.8)	30 (38.5)		
College degree or above	16 (20.5)	12 (15.4)		
***Disease type, n(%)***			3.138	0.208
Myocardial infarction	44 (56.4)	38 (48.7)		
Angina pectoris	19 (24.4)	29 (37.2)		
Myocardial ischemia	15 (19.2)	11 (14.1)		
Hypertension, n(%)	66 (84.6)	61 (78.2)	1.059	0.303
Diabetes, n(%)	20 (25.6)	14 (17.9)	1.354	0.245
***Number of coronary artery lesions, n(%)***			0.428	0.513
1	45 (57.7)	49 (62.8)		
2	33 (42.3)	29 (37.2)		

Before the intervention, there were no statistically significant differences in angina stability, angina attack frequency, physical activity limitation, disease cognition level, and treatment satisfaction scores between the two groups (P>0.05). After the intervention, the scores of the above indicators increased significantly in both groups and were notably higher in the observation group than in the control group (P<0.05; Table-II). These results indicate that the observation group showed greater improvement in quality of life than the control group.

**Table-II:** Comparison of scores between two groups of Seattle Angina Questionnaire.

Variables	Observation group (n=78)	Control group (n=78)	t/Z	P
Before intervention				
Stable state of angina pectoris	45 (41-48)	46 (41-50)	-	0.327
Frequency of angina attacks	56.7±8.5	58.3±9.3	-1.069	0.287
Restricted physical activity	52.6±5.2	51.6±5.2	1.298	0.196
Disease awareness level	30±4	30.9±4.4	-1.407	0.161
Satisfaction level of treatment	39 (37-42)	41.5 (36-45)	-	0.138
After intervention				
Stable state of angina pectoris	83.5 (77-87)^#^	75 (69-81)^#^	-	<0.001
Frequency of angina attacks	78.9±8.6^#^	73.4±7.6^#^	4.206	<0.001
Restricted physical activity	75.7±6.4^#^	65.2±10.1^#^	7.756	<0.001
Disease awareness level	57.3±6.9^#^	50.1±5.2^#^	7.31	<0.001
Satisfaction level of treatment	78 (69-82)^#^	68 (62-75)^#^	-	<0.001

***Note:*** Compared with before treatment in the same group,

# P<0.05; -, Mann-Whitney U test.

The length of hospital stay (LOS) in the observation group was significantly shorter than that in the control group [9 (8-10) *vs* 10 (9-12)], and the incidence rates of MACE [5 (6.4) *vs* 15 (19.2)], abdominal distension [2 (2.6) *vs* 9 (11.5)], irritability [2 (2.6) *vs* 10 (12.8)], and insomnia [3 (3.8) *vs* 13 (16.7)] were significantly lower in the observation group than in the control group (P<0.05). Table-III These findings suggest that the observation group had markedly lower incidence rates of MACE and complications than the control group.

**Table-III:** Comparison of LOS, MACE, and incidence of complications between two groups.

Variables	Observation group (n=78)	Control group (n=78)	χ2	P
LOS (days)	9 (8-10)	10 (9-12)	-	<0.001
MACE	5 (6.4)	15 (19.2)	5.735	0.017^a^
***Complication***				
Abdominal distension	2 (2.6)	9 (11.5)	4.792	0.029^a^
Irritability	2 (2.6)	10 (12.8)	5.778	0.016^a^
Low back pain	1 (1.3)	7 (9.0)	-	0.063^b^
Insomnia	3 (3.8)	13 (16.7)	6.964	0.008^a^
Urinary retention	3 (3.8)	8 (10.3)	2.445	0.210^a^

***Note:*** LOS, length of hospital stay; MACE, major adverse cardiovascular events; a, Pearson chi-square test; b, Fisher’s exact test.

After the intervention, 45 patients in the observation group were very satisfied with nursing care, 26 were generally satisfied, and seven were dissatisfied. In the control group, 33 patients were very satisfied, 28 were generally satisfied, and 17 were dissatisfied with the nursing care. The nursing care satisfaction rate of the observation group was 91.0%, significantly higher than that of the control group (78.2%) (P<0.05; Table-IV).

**Table-IV:** Comparison of nursing satisfaction between two groups.

Group	Very satisfied	Generally satisfied	Dissatisfied	Nursing satisfaction rate
Observation group (n=78)	45 (57.7)	26 (33.3)	7 (9.0)	71 (91.0)
Control group (n=78)	33 (42.3)	28 (35.9)	17 (21.8)	61 (78.2)
*χ^2^*				4.924
*P*				0.026

## DISCUSSION

The study demonstrated that nursing risk management exerted a significant effect on CHD patients undergoing PCI. It could effectively reduce surgery-related injuries and improve the quality of nursing care and patients’ quality of life. These findings are consistent with the research conclusions of Xin et al.^11^ and Tao et al.^12^ By establishing a special task force, nursing risk management enabled systematic identification and assessment of risks throughout the entire nursing process, breaking the passive response mode in traditional nursing practice. Targeted intervention measures were formulated for potential risks at different stages, including preoperative preparation, intraoperative operation, and postoperative recovery, thus achieving proactive risk prevention and control.^11^,^12^

The degree of improvement in quality of life is a core indicator of patients’ physical and mental rehabilitation after PCI, as it directly reflects the overall effectiveness of nursing interventions. The results of this study showed that, six months after surgery, scores on all SAQ dimensions among patients who received perioperative nursing risk management were significantly higher than those in the control group. This outcome was consistent with the study of Xin et al.^11^ It is plausible that perioperative nursing risk management did not merely focus on assisting with surgical operations but rather developed a full-cycle nursing model that included preoperative risk prediction, precise intraoperative interventions, and continuous postoperative care.^13^ A study conducted by Chen et al.^14^ indicated that personalized health education and psychological counseling before surgery could alleviate the anxiety and fear of the operation and reduce the impact of negative psychology on patients’ stress response. During the operation, refined monitoring and intervention were implemented at key risk points, such as hemodynamic fluctuations and vascular access safety, thereby minimizing surgical trauma’s interference with physiological functions. After surgery, nursing was combined with rehabilitation guidance and follow-up management, helping patients quickly regain their ability to perform daily activities and reduce the restrictions imposed by postoperative discomfort.^15^

In this study, the LOS in the observation group was significantly shorter than that in the control group, indicating that perioperative nursing risk management effectively accelerated patients’ rehabilitation. This was mainly attributed to refined intraoperative risk management, which reduced the incidence of postoperative complications and avoided prolonged hospital stays, consistent with the findings of Sugiharto et al.^16^ By optimizing puncture techniques, standardizing compression hemostasis, and enhancing monitoring of vital signs and puncture sites during the perioperative period, nursing risk management significantly reduced complications, including bleeding, hematoma, and vasovagal reflex, thereby providing a foundation for early mobilization and rehabilitation.^17^

The results of this study showed that the incidence of MACE, abdominal distension, irritability, and insomnia in the observation group were significantly lower than those in the control group, confirming the significant effect of nursing risk management in preventing and controlling complications. A study by Liu J et al.^18^ also showed that after implementing nursing risk management, the incidence of adverse cardiac events decreased significantly, from 26.51% to 8.51%. Through preoperative risk stratification, intraoperative risk management nursing accurately identifies high-risk groups (such as elderly patients, patients with hypertension, diabetes, or multivessel coronary lesions, and patients with multivessel lesions) and formulates targeted prevention and control plans to achieve precision risk management.^19^,^20^

Nursing care satisfaction reflects patients’ recognition of the professionalism, relevance, and humanistic care provided by nursing services.^21^ Undoubtedly, improved quality of life, shorter hospital stays, and lower incidence of complications lead to markedly higher satisfaction with nursing care. In this study, the nursing care satisfaction rate of the observation group was significantly higher than that of the control group. This improvement may be related to more standardized nursing procedures, timely risk communication, psychological support, and rehabilitation guidance. It is likely that this result was due to the ability of intraoperative risk management nursing to organically integrate precision intervention and humanistic care, not only meeting patients’ needs for medical safety but also taking into account their psychological and emotional needs.^22^ Compared with traditional nursing, which is characterized by uniform service delivery, the observation group received more targeted, proactive nursing care that better addressed patients’ core needs during diagnosis and treatment.^23^

The findings in this study demonstrated the promising role of nursing risk management model in the care of patients undergoing PCI, enhancing the evidence supporting the application of nursing risk management in clinical practice.

### Limitations:

Although the study has a well-defined methodology and validated measurement tools, it still has some limitations. Firstly, this was a single-center retrospective study, which introduced selection bias and affected the objectivity and representativeness of the research results. Secondly, the study’s follow-up period was relatively short. Although patients were followed for six months, the follow-up period remained relatively short, and longer-term outcomes such as sustained quality of life improvement, recurrence of complications, and durability of nursing care satisfaction could not be fully evaluated. Thirdly, the intervention measures of nursing risk management lacked quantitative standards. For example, the duration of health education and the frequency of abdominal massage were established based on clinical experience without standardized norms, which may lead to variations in implementation intensity among nursing staff and affect the consistency of intervention effects. Fourthly, this study did not analyze the cost-effectiveness of nursing risk management. Implementing nursing risk management requires additional human and material resources, and the balance between its economic and social benefits still needs further exploration. In light of the limitations outlined, further high-quality, large-scale prospective studies with long-term follow-up should be carried out to confirm the findings of the current study.

## CONCLUSION

The application of nursing risk management in CHD patients undergoing PCI is associated with reduced incidence of postoperative complications such as abdominal distension, irritability, and insomnia, shortened length of hospital stay, and improved patients’ quality of life and nursing care satisfaction.

### Recommendations:

Future multicenter, prospective studies are needed to further verify the efficacy of this nursing approach and optimize the intervention protocol to provide higher-quality, safer nursing care for CHD patients undergoing PCI.

### Acknowledgement

DeepSeek was used to correct formatting errors and improve the grammar of the manuscript.

### Authors’ contributions:

**YZ:** Literature search, study design and manuscript writing.

**ZC and MG:** Data collection, data analysis and interpretation. Critical review.

**YZ:** Manuscript revision and validation and is responsible for the integrity of the study.

All authors have read and approved the final manuscript.
